# High-resolution analysis of gene activity during the *Xenopus* mid-blastula transition

**DOI:** 10.1242/dev.102012

**Published:** 2014-05

**Authors:** Clara Collart, Nick D. L. Owens, Leena Bhaw-Rosun, Brook Cooper, Elena De Domenico, Ilya Patrushev, Abdul K. Sesay, James N. Smith, James C. Smith, Michael J. Gilchrist

**Affiliations:** 1 Division of Systems Biology, MRC National Institute for Medical Research, The Ridgeway, Mill Hill, London NW7 1AA, UK; 2 Computer Learning Research Centre, Royal Holloway, University of London, Egham, Surrey TW20 0EX, UK

**Keywords:** Midblastula transition, Maternal zygotic transition, *Xenopus tropicalis*, Polyadenylation, Transcription, RNA-seq, Gene regulatory networks

## Abstract

The *Xenopus* mid-blastula transition (MBT) marks the onset of large-scale zygotic transcription, as well as an increase in cell cycle length and a loss of synchronous cell divisions. Little is known about what triggers the activation of transcription or how newly expressed genes interact with each other. Here, we use high-resolution expression profiling to identify three waves of gene activity: a post-fertilisation wave involving polyadenylation of maternal transcripts; a broad wave of zygotic transcription detectable as early as the seventh cleavage and extending beyond the MBT at the twelfth cleavage; and a shorter post-MBT wave of transcription that becomes apparent as development proceeds. Our studies have also allowed us to define a set of maternal mRNAs that are deadenylated shortly after fertilisation, and are likely to be degraded thereafter. Experimental analysis indicates that the polyadenylation of maternal transcripts is necessary for the establishment of proper levels of zygotic transcription at the MBT, and that genes activated in the second wave of expression, including *Brachyury* and *Mixer*, contribute to the regulation of genes expressed in the third. Together, our high-resolution time series and experimental studies have yielded a deeper understanding of the temporal organisation of gene regulatory networks in the early *Xenopus* embryo.

## INTRODUCTION

Gene expression in early animal development is regulated by several mechanisms. The translational capacity of maternal mRNAs may be enhanced by polyadenylation (Aanes et al., 2011; Dworkin et al., 1985; Paris and Philippe, 1990; Simon et al., 1992) or inhibited by deadenylation (Audic et al., 1997; Graindorge et al., 2006; Paillard et al., 1998; Varnum and Wormington, 1990). As part of the maternal-zygotic transition, zygotic transcription is activated after a specific number of cell cycles. This has been widely investigated, particularly in the frog *Xenopus*, in which large-scale zygotic transcription begins after 12 rapid, synchronous cell divisions. It is accompanied by lengthening of the cell cycle and a loss of synchrony (Newport and Dasso, 1989). Together, these phenomena are termed the midblastula transition (MBT). The timing of the MBT has been suggested to depend on the titration of a maternally deposited repressive factor by increasing amounts of embryonic DNA (Newport and Kirschner, 1982a,b). However, the observation that some genes are transcribed before the MBT (Kimelman et al., 1987; Mathavan et al., 2005; Pritchard and Schubiger, 1996; Skirkanich et al., 2011; Tan et al., 2012; Yang et al., 2002) has challenged this view, and a prevailing model now involves minor (pre-MBT) and major (at MBT) waves of zygotic transcription (Tadros and Lipshitz, 2009). Recent work in the zebrafish has shown that polymorphism in cross-bred individuals can be used to distinguish between maternal and paternal mRNAs in early embryos (Harvey et al., 2013). This has helped to clarify both transcriptional and post-transcriptional regulation around the MBT.

To obtain a more detailed picture of the onset of transcription at the MBT, we have performed fine-grained expression profiling in *Xenopus tropicalis*. Our results reveal a wave of polyadenylation of maternally deposited transcripts immediately after fertilisation. This is followed by two waves of transcriptional activation, the first starting before and continuing through the MBT, with the second an hour later. We show that polyadenylation of maternal mRNAs is required for the establishment of normal levels of zygotic transcription, and for two transcription factors activated at the MBT we identify downstream target genes that are activated in the second wave of zygotic transcription. Together, our experiments provide new insights into temporal aspects of the MBT in *Xenopus*.

## RESULTS

### Sample collection and creation of expression profiles

**Fig. 1.**
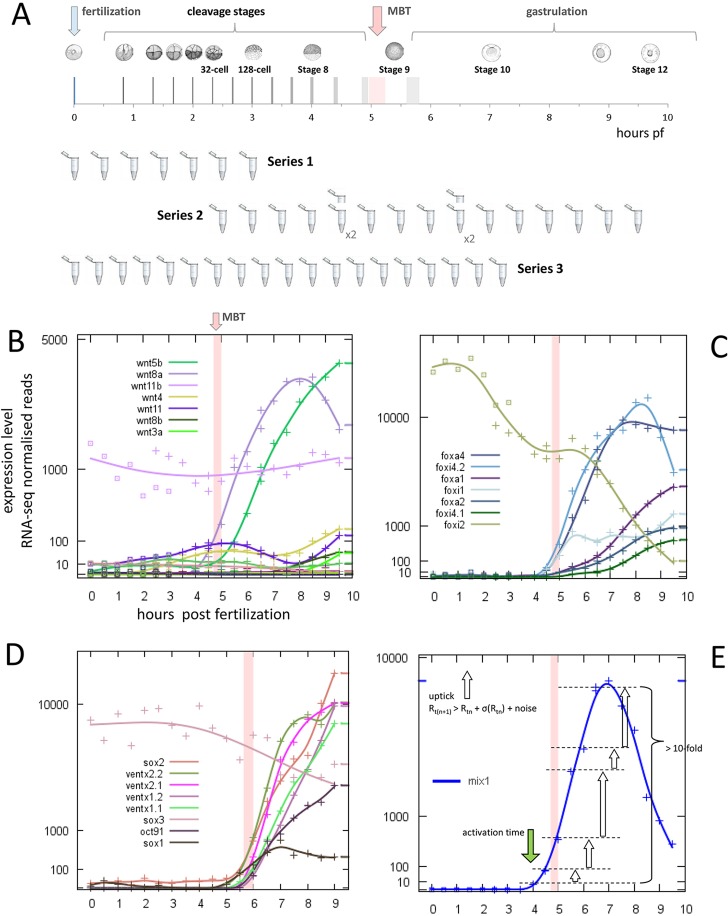
**High-resolution transcriptional profiling in early *Xenopus tropicalis* development.** (A) Embryos were sampled in groups of 50 (Series 1 and 2) or ten (Series 3) at 30-min intervals between fertilisation and early gastrulation and used to generates RNA-seq libraries for Illumina sequencing. Series 3 is scaled to the development rate of Series 1 and 2 (see text). (B-D) Expression profiles constructed from time series data. Gene families (Series 1/2): (B) Wnt; (C) Foxa and Foxi. (D) Pluripotency factors (Series 3) shown to amplify MBT in zebrafish (Lee et al., 2013). (E) Gene onset detection requires a run of successive data points, each exceeding the one before by more than is likely to have occurred by chance (see Materials and methods). Stage diagrams reproduced from Nieuwkoop and Faber (Nieuwkoop and Faber, 1994) with permission of Garland Science/Taylor & Francis, LLC.

To study the temporal organisation of gene activity in early development, we collected synchronously developing *Xenopus tropicalis* embryos at 30-min intervals between fertilisation and 9.5 h post fertilisation (hpf). These were collected in three series, each from a different female: Series 1, from 0 to 3.0 hpf; Series 2, from 2.5 to 9.5 hpf, with 4.5 and 6.5 hpf as biological replicates; and Series 3, from 0 to 9.0 hpf (Fig. 1A; Materials and Methods). Cell cycle times during the rapid cleavage stages were 20 min for Series 1 and 2, and 25 min for Series 3, placing MBT just before 5.0 hpf in the Series 1/2 data and just after 6.0 hpf in Series 3.

Illumina RNA-seq polyA+ libraries were constructed from each sample in Series 1 and 2. Series 3 samples were split after total RNA extraction, and used to make polyA+ (all samples) and ribosomal depleted Ribo-Zero libraries (whole-hour time points up to 8.0 hpf). Illumina sequence reads were mapped against the *X. tropicalis* transcriptome to generate gene counts (Materials and Methods).

Read counts were normalised by total mapped reads per library, and expression profiles were constructed from these data (Materials and Methods). These can be accessed at GEO, under accession number GSE56242, and at http://genomics.nimr.mrc.ac.uk/apps/profiles/. Data can be interrogated by gene name (Fig. 1B-D) or by expression onset time (see below).

The expression profiles were used to identify genes showing rapid and sustained changes in transcript levels. To find these, we required that there should be a run of at least three successive time points at which the point-to-point rise, or fall, in expression level was more than would be expected by chance. We defined an onset time as the first of the time points, and calculated the fold change over the run (Fig. 1E; Materials and Methods). Unless otherwise stated, onset times reported in this work will have an associated fold change ≥10. Lists of genes with onset times and fold changes are in supplementary material Table S1. The polyA+ and Ribo-Zero profiles were used together to discriminate between changes in the adenylation status of mRNAs and changes of mRNA level brought about by transcription or degradation (see below).

Overall, our RNA-seq time courses contained data points for between 17,851 (Series 3) and 21,751 (Series 1 and 2) genes. We first analyse the consistency and reproducibility of the data.

### Consistency and reproducibility

**Fig. 2.**
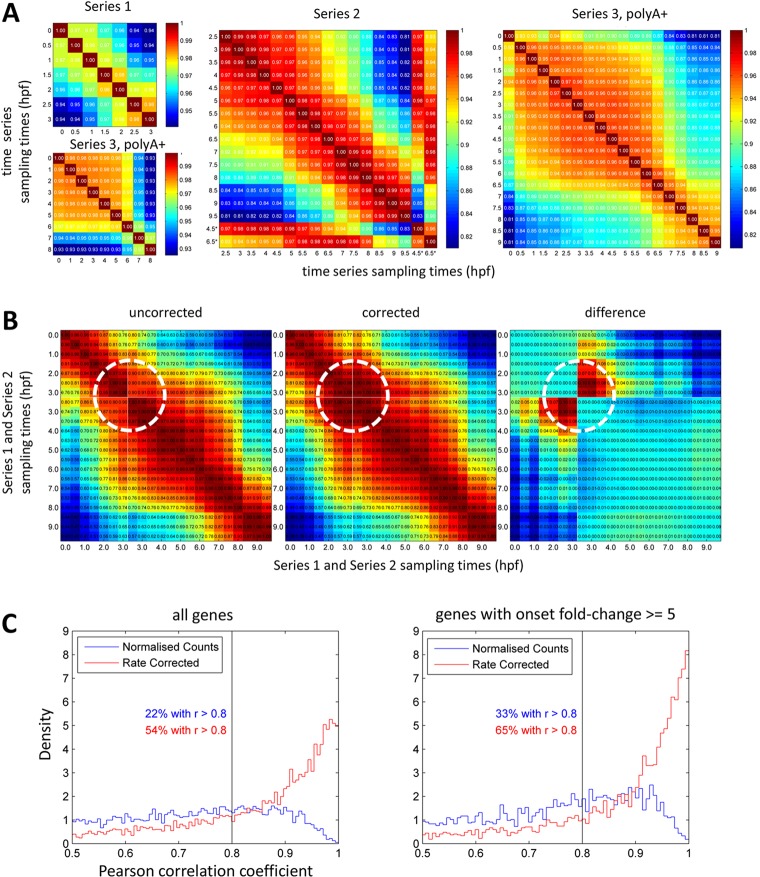
**Consistency and reproducibility in time series data.** (A) Pairwise Spearman correlation between all samples for each series; asterisks mark replicates in Series 2 (4.5 and 6.5 hpf). We chose ranked Spearman correlations over Pearson correlations as they reveal better the biological structure in the data. (B) Pairwise Spearman correlation between Series 1 and 2 samples for genes with onset fold change ≥5 validates per gene correction to join time series (see text). Circles mark cross-correlations around join. (C) Histograms of Pearson correlation coefficients between genes in Series 1/2 joined data and Series 3. Left: all genes with median normalised read count >10; right: genes with onset fold change ≥5 in either series. Blue, compared by actual sampling times; red, compared with Series 3 development rate correction.

High-resolution sampling has the benefit that neighbouring points act as replicates. This can be seen in the Pearson correlation coefficients shared between all adjacent time points over the three series (0.98±0.014, mean±s.d.), which compare favourably with the Pearson correlations of 0.98 and 0.94 between the Series 2 biological replicates at 4.5 and 6.5 hpf, respectively (Fig. 2A). This high degree of internal consistency within each time series suggests that the data will accurately reflect the underlying processes being tracked in each clutch of embryos.

**Fig. 3.**
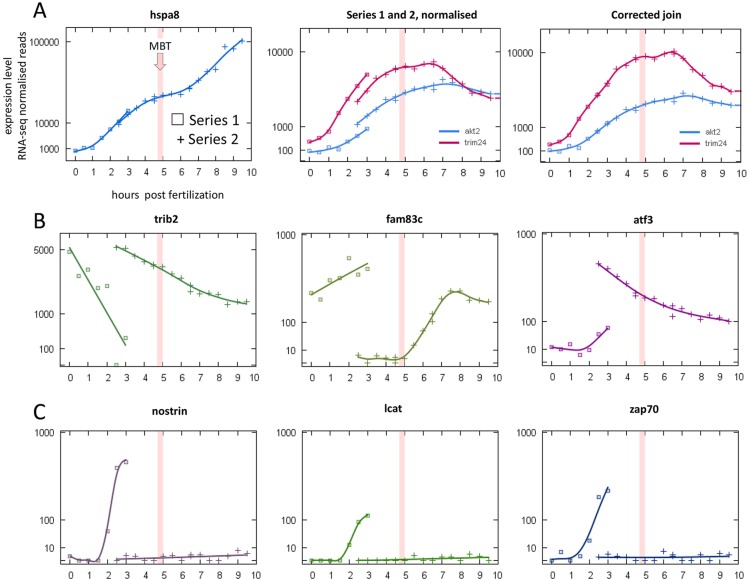
**Expression profiles showing variability in normalised maternal mRNA levels.** Study of overlapping data points between Series 1 (squares) and Series 2 (crosses). (A) Almost clean joins (left) and small offsets between series (middle) corrected with a per gene adjustment (right) (see text). (B,C) Highly divergent expression profiles suggest real variability in underlying populations of maternal mRNAs between clutches (from different mothers).

In considering consistency between series, we first explored the relationship between Series 1 and 2 within their overlap. The expression profiles of some genes failed to join cleanly, although the trends agreed (Fig. 3A). This is likely to be an effect of relative normalisation. We removed the bias by applying ‘per gene’ correction factors (Materials and Methods), which improved the correlation surrounding the join to the same level as a single fertilisation (Fig. 2B; Fig. 3A). This validated the creation of a combined Series 1/2 containing 20,661 of the 21,751 genes in the separate Series 1 and 2, and increased the number of onsets detected from 1386 to 1758. We noted that some of the genes excluded from the joined series showed highly divergent expression profiles in the separate series (Fig. 3B,C). Henceforth, Series 1/2 will refer to the joined series, whereas Series 1&2 will refer to data derived from the combined Series 1, Series 2 and Series 1/2.

We next compared the joined Series 1/2 data with the Series 3 data by computing Pearson correlation coefficients between them over the expression profiles for each gene. These correlated best when the Series 3 data were corrected for developmental rate (Materials and Methods), which raised the number of genes sharing correlation coefficients >0.8 from 33% to 65% (Fig. 2C).

**Fig. 4.**
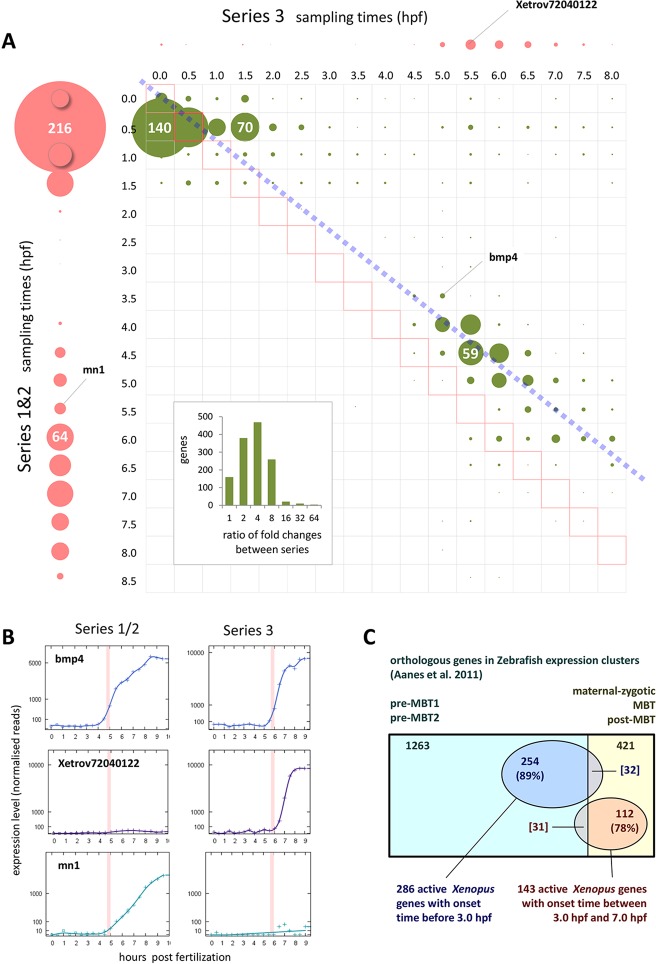
**Reproducibility and conservation in time series data.** (A) Reproducibility of onset times between Series 1&2 and Series 3. Genes with an onset time and at least a tenfold change in one or both series plotted in circles of size proportional to the numbers of genes (figures shown in white for scale). Single gene circles are not plotted. Green circles represent genes with onset fold change of at least 2× in the other time series, allowing for significant difference in fold change (see text). Genes in circles adjacent to dashed blue line (developmental time equivalence between Series 1&2 and Series 3; see Materials and methods) are considered to have reproducible onsets. Pink circles represent genes having detectable onset in only Series 1&2 (vertical axis) or Series 3 (horizontal axis). Inset: numbers of genes with onsets in both series at different ratios of fold change. (B) Examples of genes (marked in A) showing: reproducible onset times (*Bmp4*); no detectable onset in Series 1&2 (*Xetrov72040122*); and no detectable onset in Series 3 (*Mn1*). (C) Conservation shown in overlap between *Xenopus* and zebrafish genes associated with either polyadenylation or transcription. Numbers show frog-fish orthologues; genes in zebrafish expression profile clusters (Aanes et al., 2011) shown in rectangles, and *X. tropicalis* genes with onset times indicating polyadenylation or transcription (fold change threshold ≥5) shown in ovals.

To assess reproducibility of onset times, we took all genes with an onset in either or both of the Series 1&2 or Series 3 data, of which there were 2141. For those with an onset in only one set of data we allowed a fold change below the threshold in the other: this lower fold change had to be at least twofold, and could not be less than one-tenth of the higher fold change in the other time series. This gave us 1228 genes with detectable onset times in both sets of data, and these we found to be highly reproducible (Fig. 4A,B, upper panel), although the fold changes were less so (Fig. 4A, inset). In addition, these data confirm the developmental delay of Series 3 predicted from the cleavage stage cell cycle times (Fig. 4A). Reproducibility of onset times between data sets was highest in the later cleavage stages and around the MBT, and somewhat lower in the early cleavage stages.

Overall, we found consistency within time series and reproducibility between them, especially with regard to onset times; however, we also found significant differences in the behaviour of some genes.

### Rising polyA+ transcript levels: successive waves of activity

**Fig. 5.**
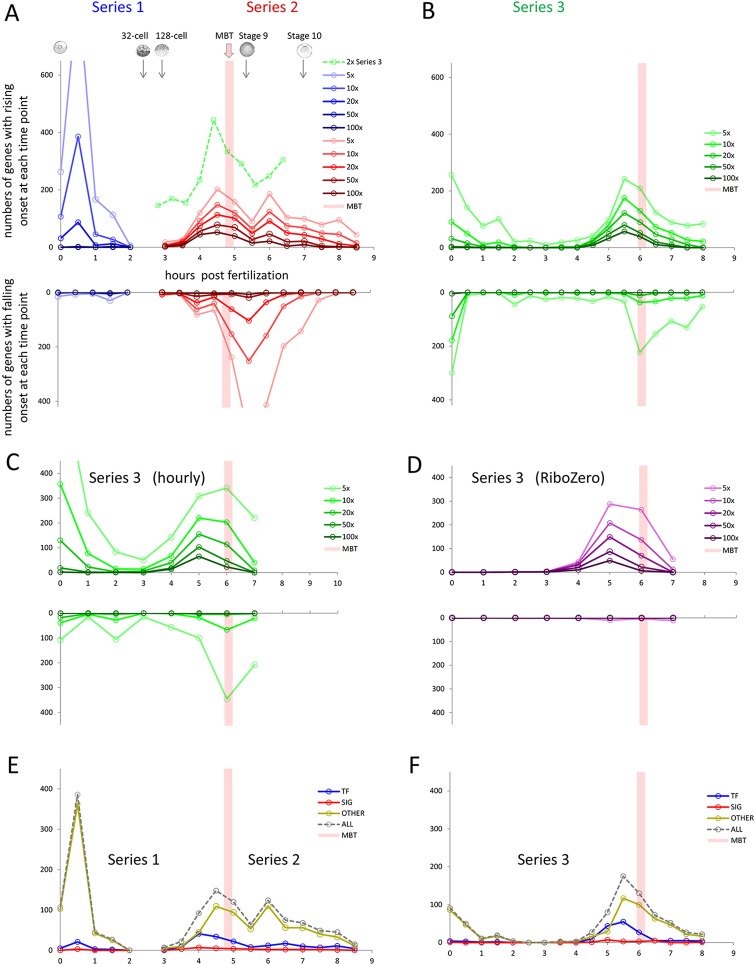
**Gene activity profiles showing successive waves.** Each plot shows the number of genes with onset times at each sampling point in the various time series, showing the data for different fold change thresholds (see text). Positive vertical axes represent onset of rising transcript levels, negative axes represent onset of falling levels. All data are plotted against actual sample times, except the 2× Series 3 in A, which is corrected for development rate (Materials and Methods). MBT (pink bar) gives consistent developmental time scale. (A) PolyA+ Series 1 and 2. Also shown is time-adjusted Series 3 data using a more sensitive end-of-series 2× threshold (Materials and Methods) to show detection of the third wave in this developmentally shorter time series. (B) PolyA+ Series 3. (C) PolyA+ Series 3 analysed at one-hour intervals. (D) Ribo-Zero Series 3. (E,F) Activity profiles using 10× threshold, organised by gene type: TF, transcription factor; SIG, signalling molecules and receptors; other, genes not classified as TF or SIG. Stage diagrams reproduced from Nieuwkoop and Faber (Nieuwkoop and Faber, 1994) with permission of Garland Science/Taylor & Francis, LLC.

As outlined above, we found 2141 genes in the three time series that showed a sustained increase in polyA+ transcript levels. These had onset times distributed in three waves of activity between fertilisation and early gastrulation: the first between fertilisation and the 64-cell stage, the second from the 128-cell stage through MBT to stage 9, and the third from stage 9 to shortly before stage 10 (Fig. 5A, upper). The timing of the waves was reproducible, although the third wave was only weakly detected in the developmentally shorter Series 3. The numbers of genes in the first wave differed between Series 1 and Series 3 (discussed below). The gap in the activity profiles between Series 1 and Series 2 was confirmed in the Series 3 data to contain few onset times. We observed that the onset fold changes of genes in the first wave were generally smaller than those in the second and third waves (Fig. 5A,B).

### Characterisation of the waves

**Fig. 6.**
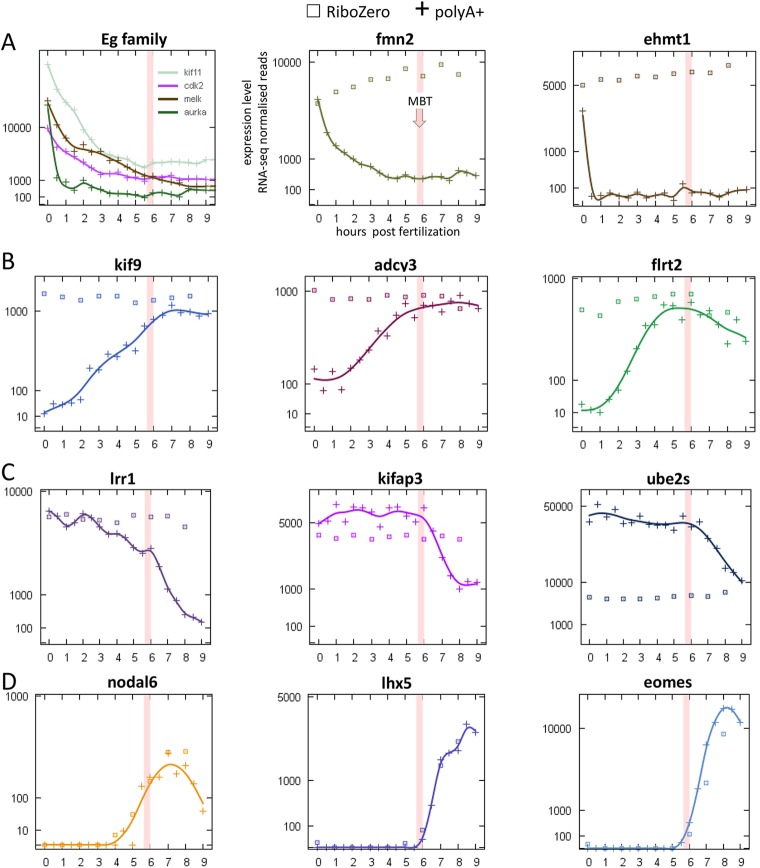
**Expression profiles of split sample polyA+/Ribo-Zero RNA-seq data illustrating mechanisms underlying changes in polyA+ transcript levels.** Data from Series 3: squares, Ribo-Zero; crosses, polyA+. (A) Eg family of genes: controls for detection of post-fertilisation deadenylation of maternal mRNAs; and other deadenylated genes. (B) Post-fertilisation polyadenylation of maternal mRNAs. (C) Deadenylation of maternal mRNAs at MBT. (D) Transcriptional activation of genes with onset time in the second wave.

To distinguish between zygotic transcription and polyadenylation of maternal mRNAs, we used the hourly time points in Series 3 to compare polyA+ and Ribo-Zero data (Fig. 5C,D, upper). Before 2.0 hpf, we found no genes with onset times in the Ribo-Zero data, compared with 434 in the polyA+ data, indicating that the rapid increases seen in the polyA+ data are due to polyadenylation of maternal mRNA and not to transcription (Fig. 6B). After 3.0 hpf, the polyA+ and Ribo-Zero data become similar, indicating that transcription is now predominant (Fig. 6D).

**Fig. 7.**
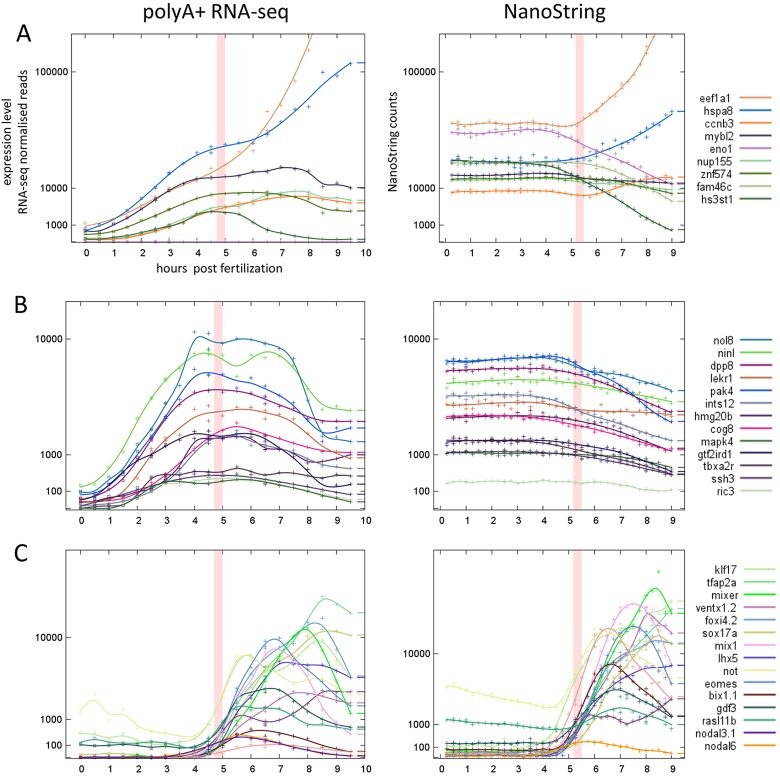
**Comparison of polyA+ and total mRNA expression profiles confirming underlying mechanisms.** Although the polyA+ mRNA and NanoString total RNA data are taken from different clutches, the two data sets are entirely consistent. (A,B) Higher and lower expression level genes with onset times before 3.0 hpf showing polyadenylation of maternal mRNAs. (C) Genes with onset times after 3.0 hpf showing zygotic transcription.

This was independently confirmed by measuring total mRNA levels with the NanoString nCounter (Geiss et al., 2008). Our test set contained 22 early-onset genes (before 3.0 h in Series 1/2), and 17 late-onset genes (at or after 3.0 hpf in Series 1/2). Embryos were collected at 15-min intervals from fertilisation until 9.0 hpf and the resulting NanoString counts were normalised against *Odc1* (Materials and Methods). Genes in the early-onset group showed constant levels of total mRNA over the first 3 hours while their polyA+ levels were rising, consistent with polyadenylation of maternal mRNAs (Fig. 7A,B). Genes in the late-onset group showed low levels of total mRNA initially, rising synchronously with the polyA+ data after 3.0 hpf, consistent with zygotic transcription (Fig. 7C).

In summary, between fertilisation and the 64-cell stage we observe a wave of polyadenylation of maternal mRNAs. This is followed by a continuous broad wave of transcriptional activation, from the 128-cell stage and extending through the MBT to the start of gastrulation at stage 9. This is closely followed by another wave of transcriptional activation lasting about 1 h and diminishing before stage 10. Characterising the waves from Series 1 and 2 data (Fig. 5E), we found that the first wave contained 551 genes, of which 28 (5%) were transcription factors and three were signalling molecules (Materials and Methods). The second wave contained 409 genes, of which 113 (27%) were transcription factors and 20 (5%) were signalling molecules. The third wave contained 213 genes, of which 30 (14%) were transcription factors and five were signalling molecules. GO analysis of the three waves is presented below and in Table 1.

**Table 1.**
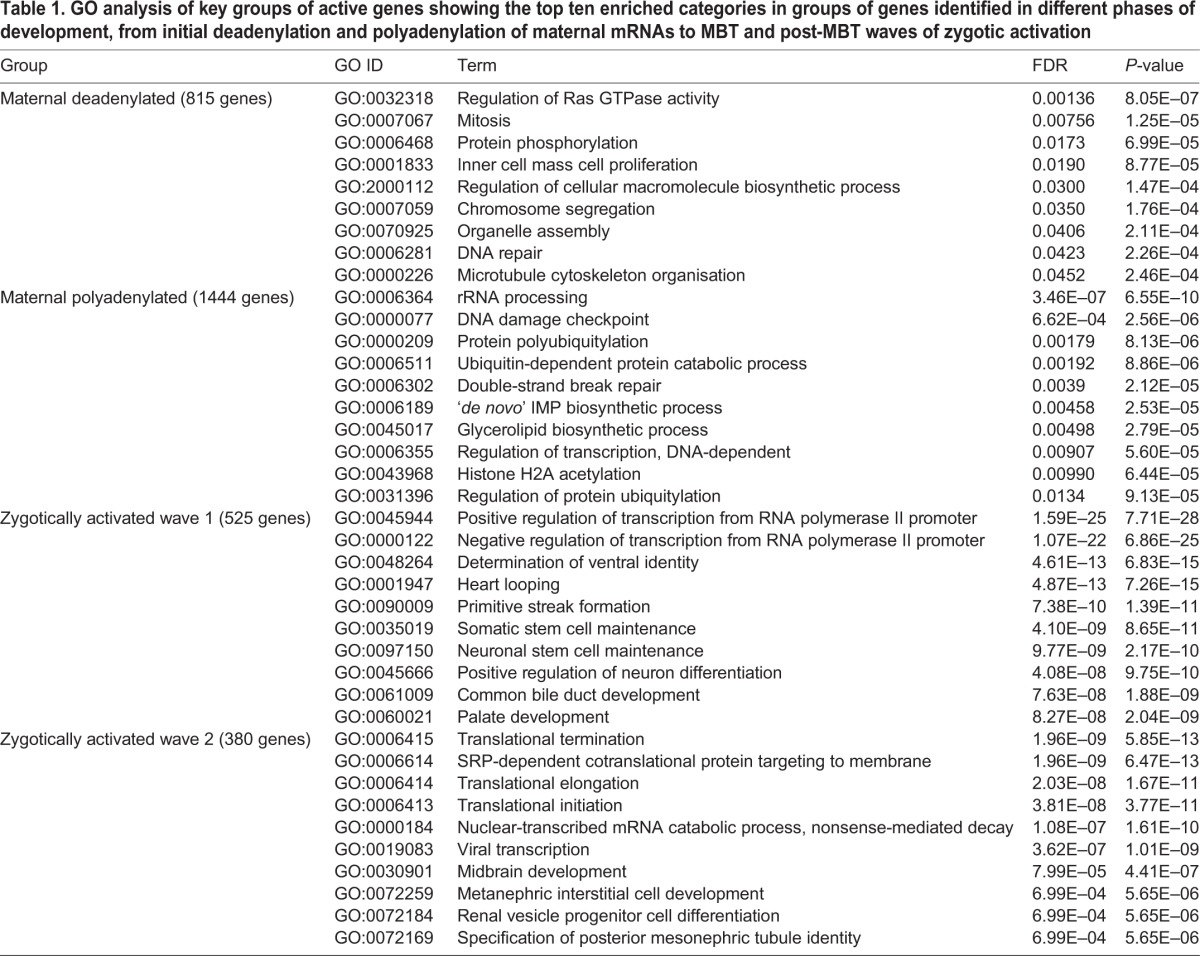
**GO analysis of key groups of active genes showing the top ten enriched categories in groups of genes identified in different phases of development, from initial deadenylation and polyadenylation of maternal mRNAs to MBT and post-MBT waves of zygotic activation**

There are more genes (551) in the polyadenylation wave than in the zygotic activation wave (409). This is consistent with data from zebrafish, in which more genes were found in clusters associated with polyadenylation of maternal mRNAs than with transcriptional activation (Aanes et al., 2011). We note that the Series 3 data suggest that the balance between maternal polyadenylation and zygotic transcription may be variable (Fig. 5A,B, upper).

### Falling polyA+ transcript levels: deadenylation and degradation

We found 1092 genes in the three time series that showed a sustained decrease in polyA+ transcript levels. There were two periods of activity, the first before the 64-cell stage and the second at MBT (Fig. 5A,B, lower). Interestingly, the earlier activity is much less obvious in Series 1 (ten genes) than in Series 3 (193 genes). Comparison of the polyA+ and Ribo-Zero data over the first period in Series 3 (Fig. 5C,D, lower) indicates that the decrease is due to deadenylation rather than degradation. To explore this idea further, we looked at the Eg family of genes (*Cdk2*, *Aurka*, *Melk*, *Kif11*), which are known to be deadenylated after fertilisation (Paillard et al., 1998): all showed significant loss of adenylated transcripts, albeit at somewhat different rates (Fig. 6A). The most rapid was *Aurka* (*Eg2*), which approached maximum deadenylation before the end of the first cell cycle. We found a further 234 genes showing similar behaviour to *Aurka* in the Series 3 data (e.g. *Ehmt1* in Fig. 6A), although we note that this conclusion depends, for each gene, on a single data point.

The Series 3 polyA+ data showed onset of rapid loss of several hundred genes at MBT (Fig. 5C, lower). The corresponding RiboZero data (Fig. 5D, lower) showed no onsets, indicating that loss of polyA+ transcripts in this period was also by deadenylation (Fig. 6C).

### Gene ontology (GO) analysis

We performed GO analysis (Materials and Methods) on four groups of genes showing at least a fivefold change in expression (a fivefold rather than tenfold threshold was chosen to increase the number of genes available for analysis): (1) deadenylated maternal transcripts; (2) polyadenylated maternal transcripts; (3) the first wave of zygotic transcripts; and (4) the second wave of zygotic transcripts. Table 1 lists the top ten categories in each group, with full lists in supplementary material Table S2. Interestingly, the maternal deadenylated group (in which protein synthesis is likely to be reduced) is enriched for genes involved in mitosis and DNA repair, whereas the maternal polyadenylated group (in which protein synthesis is likely to be increased) is enriched for genes involved in rRNA processing, DNA damage checkpoint and ubiquitylation. The first wave of zygotic activation is enriched for genes involved in regulation of transcription, stem cell maintenance and axis patterning and development, whereas the second wave of zygotic activation is enriched for genes involved in control of translation, and the early signs of organogenesis, in heart and kidney development.

### Comparison with zebrafish

To study the conservation of these waves of activity, we compared our results with work in the zebrafish (Aanes et al., 2011), in which 3039 polyadenylated maternal transcripts and 1253 zygotically activated genes were identified. The zebrafish study was performed and analysed differently, and to facilitate comparison we used a fivefold threshold in our data. Of the 3039 polyadenylated zebrafish transcripts, 1263 had orthologues (according to assigned gene names) in our *X. tropicalis* reference data. Of these, 285 have onset times up to 7.0 hpf in our expression profiles, with 254 (89%) occurring before 3 h. Of the 1253 zebrafish zygotic genes, 421 had orthologues. Of these, 144 have onset times up to 7.0 hpf, with 112 (78%) occurring between 3.0 hpf and 7.0 hpf (Fig. 4C). Similar results were obtained for Series 3 data (not shown). The overlap between these different vertebrate species suggests not only that the general pattern of activity is conserved, but also that the timing of the roles of individual genes in development is conserved.

### Significance of the first wave of polyadenylated maternal mRNAs

**Fig. 8.**
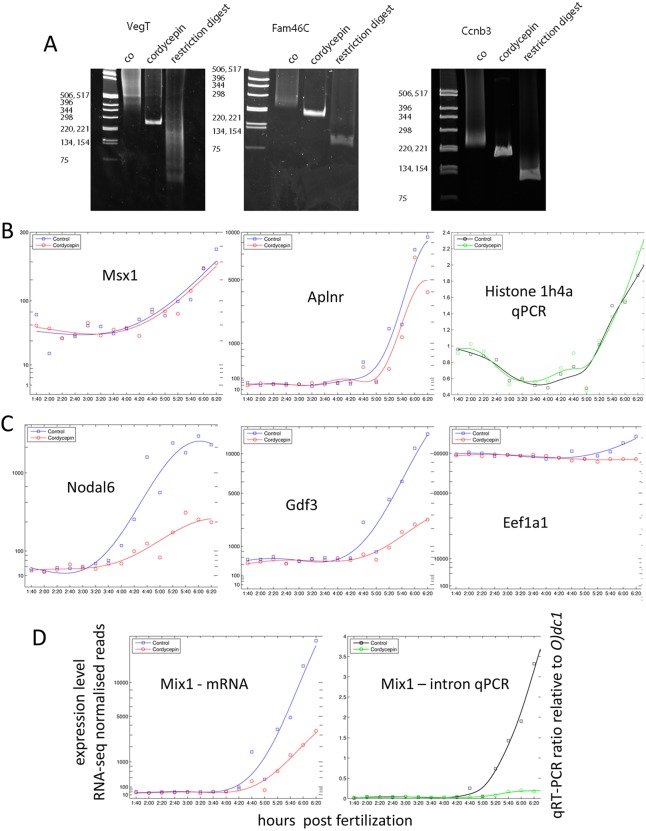
**Variable transcriptional deficit at MBT in embryos treated with cordycepin.** (A) Confirmation of inhibition of polyadenylation: shortened polyA+ tail lengths in cordycepin-treated embryos for the polyadenylated maternal genes *VegT*, *Fam46c* and *Ccnb3*. Lanes: ‘co’, long and variable polyA+ tail in controls; ‘cordycepin’, consistently short polyA+ tail in treated embryos; ‘restriction digest’, confirmatory restriction digest of a gene-specific PCR fragment (Materials and Methods). (B-D) Transcriptional activation of genes in cordycepin-treated versus control embryos by NanoString nCounter (red/blue) or qPCR (green/black). (B) Transcription unaffected by cordycepin treatment. (C) Substantially reduced transcription levels after cordycepin treatment. (D) Loss of primary transcripts for *Mix1* shown by intron qPCR confirms loss of transcription, not degradation of deadenylated transcripts.

Having established that the polyadenylation of maternal transcripts is conserved, we explored its significance in establishing normal levels of zygotic transcription at MBT. To do this, we made use of cordycepin, a modified form of adenosine that is a potent inhibitor of polyadenylation (Aoki et al., 2003), and which effectively inhibited polyadenylation of *VegT*, *Fam46c* and *Ccnb3* in *Xenopus tropicalis* (Fig. 8A; Materials and Methods). To determine whether cordycepin affects the expression of genes at MBT, we selected 42 genes with onset times in the early part of the second wave (3.0 to 4.0 hpf) and 24 in the later part (4.5 to 5.0 hpf) and analysed their expression profiles using NanoString nCounter.

Two genes in the test set, *Aplnr* and *Msx1*, were largely unaffected by cordycepin (Fig. 8B). The majority of the remainder were transcribed, but expression was reduced up to tenfold compared with controls (Fig. 8C,D), suggesting that polyadenylation of maternal transcripts is necessary to achieve the correct levels of gene activation at the MBT.

We were concerned that cordycepin may have a general inhibitory effect on transcription (Shiokawa and Misumi, 1983; Siev et al., 1969), although the normal expression of *Aplnr* and *Msx1* argues that this is not the case. To provide further evidence for this view, we performed qRT-PCR on *Histone 1h4a*, the mRNA of which is not polyadenylated (Ballantine and Woodland, 1985) and should therefore be resistant to any non-specific effect of cordycepin. *Histone 1h4a* was expressed and activated at the same levels as in controls (Fig. 8B).

To confirm that the decrease in expression of genes in the second and third waves in response to cordycepin was due to a decrease in transcription and not to the degradation of non-polyadenylated transcripts caused by residual cordycepin, we investigated levels of unspliced *Mix1* RNA, using intron-specific primers (Materials and Methods). Levels of unspliced transcripts were markedly reduced in cordycepin-treated embryos compared with the controls (Fig. 8D).

Together, our observations are consistent with the idea that the failure to fully polyadenylate some maternal mRNAs reduces the transcription levels of many genes at the MBT.

### Relationship between transcriptionally activated genes in the second and third waves

**Fig. 9.**
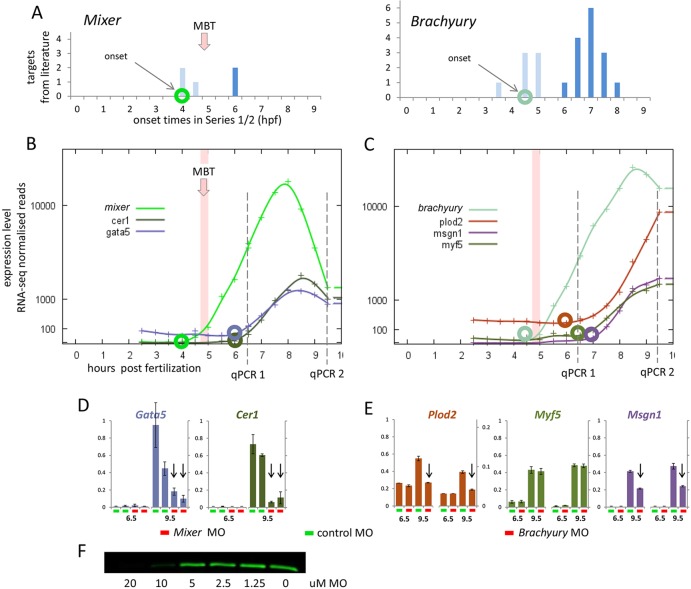
**Targets of MBT-activated transcription factors are found activated within 1-2 h in the third wave.** (A) Onset times from Series 2 data for published targets of transcription factors *Mixer* (Loose and Patient, 2004; Sinner et al., 2006) and *Brachyury* (Gentsch et al., 2013). Numbers of targets shown at each time point: light blue, onset in second wave; dark blue, onset in third wave or beyond. Onset time of transcription factor is indicated by circle on time axis. (B,C) Expression profiles of *Mixer* and *Brachyury*, and published targets selected for qPCR. Circled data points show onset times, dashed lines indicate qPCR sample points. (D,E) qPCR data relative to *Odc1* for selected targets at 6.5 hpf and 9.5 hpf, comparing expression in transcription factor morpholino (MO)-injected versus control MO-injected embryos. Arrows show reduced expression in transcription factor knockdown. Data are two technical replicates from pairs of biological replicates: *Mixer* biological replicates are from a single clutch, *Brachyury* biological replicates are from different clutches. (F) Inhibition of *in vitro* translation of *Mixer* by *Mixer* MO showing the threshold concentration of morpholino able to block the translation of HA-tagged *Mixer*.

Next, we explored the possibility that transcription factors activated in the second wave might regulate the expression of genes activated in the third wave. To do so, we chose two genes activated in the second wave the putative targets of which we could infer from a meta-analysis of gene regulatory networks in early *Xenopus* development (Loose and Patient, 2004), as well as from specific studies of the two genes in question: *Mixer* (Sinner et al., 2006) and *Brachyury* (Gentsch et al., 2013). The putative targets selected were *Cer1* and *Gata5* for *Mixer*, and *Plod2*, *Msgn1* and *Myf5* for *Brachyury* (Fig. 9A-C).

As expected, none of the putative targets was expressed at 6.5 hpf in embryos injected with the appropriate transcription factor antisense morpholino oligonucleotides (Fig. 9D,E). At 9.5 hpf, expression of both *Cer1* and *Gata5* was downregulated in the *Mixer* morpholino-injected embryos relative to the controls (Fig. 9D), and expression of *Plod2* and *Msgn1* was similarly downregulated in the *Brachyury* morpholino-injected embryos (Fig. 9E). This is consistent with the idea that these genes are being transcriptionally activated, in the third wave, by *Mixer* and *Brachyury*, respectively. Expression of *Myf5* was not affected by loss of *Brachyury* (Fig. 9E), but co-injection of additional antisense morpholino oligonucleotides targeting the related T-box genes *Eomes* and *zVegT* caused expression of *Myf5* to be reduced significantly at 9.5 hpf (data not shown). Work by Gentsch and colleagues has shown that the T-box genes regulate gene expression cooperatively in the early embryo of *X. tropicalis* (Gentsch et al., 2013).

We conclude that some genes in the third wave are downstream targets of transcription factors that are activated in the second wave. The short time interval between the activation of *Mixer* and *Brachyury* and their target genes suggests that the targets are direct, and for the *Brachyury* targets this conclusion is supported by chromatin immunoprecipitation peaks found in the vicinity of the target genes (Gentsch et al., 2013).

## DISCUSSION

In this work, we describe the pattern and sequence of gene activity in the *Xenopus tropicalis* embryo between fertilisation and the onset of gastrulation. A distinctive feature of this work is the use of a fine-grained and temporally unbiased embryonic time series. The high time resolution has allowed us to observe changing transcript levels, and hence determine onset times with some degree of precision. Replication of our time series data has allowed us to show that the timing of transcriptional activation is reproducible between independent experiments (Fig. 4A), which suggests that activation times may provide a better basis for understanding gene regulatory activity than would changes in transcript levels between more widely separated time points.

An important result has been the estimation of the interval between activation of transcription factors and activation of their downstream targets. Combined with onset times, these provide useful constraints within which to evaluate existing gene regulatory relationships, and aid the interpretation of relationships in future work.

Replication also allowed us to explore some striking inconsistencies, both in the behaviour of individual genes (Fig. 3B,C; Fig. 4B) and in the general adenylation status of maternal mRNAs after fertilisation. Series 1 showed fewer genes undergoing deadenylation than Series 3 (Fig. 5A,B, lower) and more genes undergoing polyadenylation (Fig. 5A,B, upper). This suggests that maternal mRNAs in embryos sampled for Series 3 were more highly polyadenylated prior to fertilisation, requiring both less polyadenylation of some transcripts and more deadenylation of others, relative to Series 1. Although variability in this system could be explored in more detail with further replicates of the time series, we feel that we are able to reach sound conclusions regarding the temporal organisation of gene activity around the MBT based on the data we have described.

Recent work in the zebrafish has used ribosomal profiling to identify highly translated maternal transcription factors, and has highlighted *Pou5f1*, the *SoxB1* family and *Nanog*, for which combined loss of function causes decreased expression in 74% of zygotically transcribed genes (Lee et al., 2013). Interestingly, only one of these (*Sox3*) has significant levels of maternal transcripts in *X. tropicalis* (Fig. 1D). Of the others, the *Xenopus* orthologue of *Pou5f1* (*Oct91*) cannot be detected maternally and is clearly activated at MBT. No *Xenopus* orthologue of *Nanog* has yet been identified, and although the homeobox transcription factors *Ventx1/2* have been shown to function in a *Nanog*-like manner (Scerbo et al., 2012), none of these is detected as maternal transcripts, and all are strongly activated at MBT. *Sox1* and *Sox2* were both detected at low levels maternally before being strongly activated at MBT (Fig. 1D).

Our findings with respect to waves of activity differ from those of Tan et al. (Tan et al., 2012) who interpreted increasing levels of specific polyA+ transcripts between the 2-cell and 32-cell stages in *X. tropicalis* as representing genes for which transcription is activated before the MBT. Furthermore, given that we observe a continuous broad wave of zygotic transcription, commencing in the rapid cleavage stages before MBT and finishing after it, we find no evidence to support the notion that zygotic transcription around the MBT is divided into minor (pre-MBT) and major (MBT) waves (Tadros and Lipshitz, 2009). Our data are, however, consistent with results in *X. laevis* showing an increase in transcript levels starting between the 4-cell and 256-cell stages (Skirkanich et al., 2011), and with earlier work reporting low levels of transcription from the 128-cell stage (Kimelman et al., 1987). Overall our high-resolution time series and experimental studies have yielded a deeper understanding of the temporal organisation of gene regulatory networks in the early *Xenopus* embryo.

## MATERIALS AND METHODS

### Embryo collection and library preparation

Embryos of *Xenopus tropicalis* were obtained as described (Khokha et al., 2002) and cultured at 23°C. Pools of embryos were obtained from single fertilisations at 30-min intervals. Three time series were collected: Series 1 and 2 were collected in pools of 50, and Series 3 in pools of ten (see Results for details). Embryos for Series 3 were derived and collected from a different frog colony. Cleavage times were noted up to the sixth cleavage for all fertilisations, and early- or late-cleaving embryos were excluded from analysis. For Series 1 and 2, total RNA was isolated using TriPure (Invitrogen) followed by LiCl precipitation. RNA was dissolved in 100 μl H_2_O, and 10 μg total RNA from each sample was used for PolyA+ RNA library preparation using Illumina kit RS-930-1001. For Series 3, total RNA was extracted, samples were suspended in 40 µl DEPC-treated H_2_O, and 1 μg was taken for library preparation. PolyA+ RNA was isolated using beads from TruSeq RNA Sample Prep Kit v2 (Illumina kit RS-122-2001), and PolyA+ RNA-seq libraries were prepared using Epicentre ScriptSeq v2. Ribosomal RNA-depleted RNA-seq libraries were prepared using Epicentre ScriptSeq Complete Kit (BHMR1224) combining Ribo-Zero and ScriptSeq v2.

### Illumina sequencing and data processing

Series 1 and 2 were sequenced on the Illumina GA-IIx platform using standard methods to generate 39-bp paired end reads. Series 3 was sequenced on the Illumina Hi-Seq 2500 using standard methods to generate 50-bp paired end reads. Reads were mapped using Bowtie 0.12.7 (Langmead, 2010) with parameters ‘-a -y --best -v 3 -X 0 -I 10000’ against a set of *Xenopus tropicalis* transcript sequences. This set included gene models from the v7.1 genome assembly, downloaded from Xenbase (James-Zorn et al., 2013) at ftp://ftp.xenbase.org/pub/Genomics/JGI/Xentr7.1/Xentr7_2_Stable_Transcript.fa.gz, to which we added curated sequences for missing gene models, both from assembled EST contigs (Gilchrist et al., 2004) and from NCBI cDNA sequences. The additional sequences are available as a FASTA file in the supplementary material, and also included ribosomal and mitochondrial sequences. Reads mapping to transcripts from more than one gene were discarded. Gene counts were normalised by total mapped reads per library (excluding ribosomal alignments) to a standard library size of 25 million reads (close to the mean). The raw read count 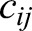 of gene 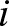 in library 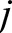 is normalised to 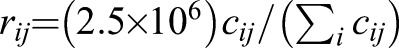.

### Comparison of data between different fertilisations

We scaled our time series to account for differences in developmental rates. After the first cleavage, the average cell cycle was 20 min in Series 1 and 2 and 25 min in Series 3. Where required, we scaled the hours post fertilisation of Series 3 by 20/25 to facilitate comparison.

We joined the Series 1 and Series 2 data by applying a per gene correction calculated from the normalised expression values in the overlap region at 2.5 and 3.0 hpf. This was calculated as: 
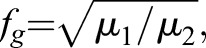
 where 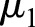 and 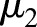 are the means from the two series in the overlap. For each gene, we shifted the two series towards each other by this factor where 
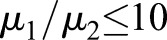
, and the two values at each point were subsequently within the range that is likely to be seen by chance (see below). The joined Series 1/2 was validated by pairwise Spearman correlation (Fig. 2B).

### Gene type identification

Transcription factors were identified using the set of Pfam DNA binding domains (Finn et al., 2010) identified in the DBD database of transcription factors (Wilson et al., 2008) with the HMMER3 program (Eddy, 2011) and an E-value threshold of 0.01. Transcription factors known to be missed by this approach were added manually (e.g. the Sox family). Signalling molecules and their receptors were identified from compiled lists of known genes. Genes not identified in either category are referred to as ‘other’.

### Empirical estimation of error in the RNA-seq data

The technical noise of RNA-seq follows a Poisson model (Marioni et al., 2008), and for any gene the variance of read counts will be greater than the mean by a factor proportional to the biological noise. We therefore used the square root of read counts for smoothing (see below) and visualisation, which serves to reduce the variance caused by sequencing. We applied a threshold of 
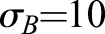
 reads where low-count noise was too dominant. From Series 2 biological replicates at 4.5 and 6.5 hpf, we calculated an empirical parameter 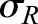, which is defined as the 0.98 quantile of differences between replicates at expression level *R*. We found that 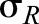 is well described by a log-linear model: 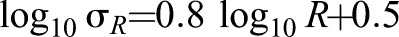. We used this relationship to identify changes in expression level that are unlikely to occur by chance.

### Smoothing of expression profiles

To improve visualisation, we applied a non-parametric method called ‘smoothing splines’ to smooth the gene expression time series (Bar-Joseph et al., 2003; Craven and Wahba, 1979; Dejean et al., 2007; Hastie et al., 2009). We fitted smoothing splines to square-rooted read counts and chose the smoothness parameter by generalised cross-validation (Craven and Wahba, 1979).

### Detection of significant changes in gene expression

We detected significant increases and decreases in transcript levels, reversing the sign for decreases. For a gene at time *t* with normalised read count 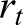, we found runs in which 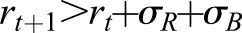 holds. That is, the expression level of a gene is rising more than would be expected by chance. We applied a run length and fold change condition: if a run starts at time index *s* and ends at time index *e* we require 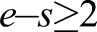 and 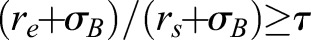 (see Fig. 1E). For the majority of data here, we used 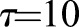. More than one onset time can be recorded for a gene. We joined two runs of at least three points that are 30 min apart if the linking points satisfied 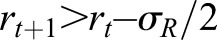. For analysis at hourly intervals, we allowed the last two time points in the data to be recorded as a run. Runs of three points were excluded if the count before or after the run was respectively greater or less than the average of the run. As Series 2 only starts at 2.5 hpf, onset times equal to 2.5 hpf in Series 2 were excluded.

To detect onsets within a time series to the same (lower) sensitivity as near the end, we re-tested iteratively by removing the last time point in each subsequent iteration. At each cycle, we kept just the onset times at two time intervals before the current last time point.

### GO analysis

The transcript set was mapped onto the NCBI non-redundant protein database for human, mouse, fly and frog proteins using the BLASTx program (Altschul et al., 1990). GO terms were assigned to the genes using the blast2go program (Conesa et al., 2005). Enrichment analysis was performed using Fisher's Exact Test with FDR cutoff value of 0.05, and limited to the ‘Biological Process’ namespace.

### NanoString confirmation of activation mechanisms

Gene probes were designed by NanoString (www.nanostring.com). Embryos were collected in groups of five at 15 min intervals, before snap-freezing in Trizol. Total RNA was extracted and samples were diluted to 50 µl before analysis (www.nucleomics.be). Data were normalised against *Odc1*. The average cell cycle length after the first cleavage was 21 min, so we corrected the timescale by 20/21, as above.

### Polyadenylation blocking in cordycepin-injected embryos

To inhibit cytoplasmic polyadenylation before MBT, embryos were injected at the one-cell stage with 200 pmol cordycepin (Sigma), or H_2_O as a control. Samples of ten embryos were collected at 3.5 hpf to confirm inhibition of polyadenylation. For time series analysis using NanoString nCounter (above), we collected cordycepin-treated and control embryos in groups of five at 20 min intervals between 1 h 40 min and 6 h 20 min after fertilisation. Total RNA was prepared as described above and analysed by NanoString.

To confirm inhibition of polyadenylation, an aliquot of 4 μg of total RNA was ligated in a volume of 10 μl for 30 min at 37°C with 0.4 μg of a 3′-amino 5′-phosphorylated oligonucleotide P1 (5′-P-GGTCACCTTGATCTGAAGC-NH_2_-3′) using T4 RNA ligase (New England Biolabs). The reaction was then placed at 70°C for 15 min. The whole 10 μl ligation reaction was used in a 50 μl reverse transcription reaction using Superscript III (Invitrogen), according to the manufacturer's directions, using 0.4 μg primer P2 (5′-GCTTCAGATCAAGGTGACCTTTTT-3′) (Graindorge et al., 2006). Of the resulting cDNA, 1 μl was used in a 50 μl PCR reaction in a 50 μl final volume. As a reverse primer, P2 was used in all reactions, with the following gene-specific forward primers: VegT, 5′-GGGGGTACAGGCAGAACAGT-3′; Fam46c, 5′- TTGACCAGATGGGTGACTGA-3′; Ccnb3, 5′-CTCTTCTC-CTTGCCCTTGTG-3′. The PCR reaction mixture contained 1× reaction buffer (Invitrogen), 0.2 μM of each primer (forward and reverse), 200 μM dNTPs, 1 U of Platinum Taq Polymerase (Invitrogen), 1.5 mM MgCl_2_. The amplification program consisted of a preincubation step (95°C for 5 min), followed by 35 cycles consisting of a denaturation step (95°C for 30 s), an annealing step (56°C for 30 s) and an extension step (72°C for 30 s), and then one final extension step (72°C for 7 min).

### cDNA preparation and qRT-PCR

Total RNA was isolated from *Xenopus* embryos using the TriPure reagent (Invitrogen) according to the manufacturer's instructions, followed by a LiCl precipitation. mRNA expression was validated by quantitative RT-PCR using the LightCycler 480 (Roche). Reverse transcription was carried out using the Transcriptor First Strand cDNA kit (Roche) followed by quantitative real-time PCR using a LightCycler 480 SYBR Green I Master Kit (Roche) following the manufacturer's instructions. Primer sequences are listed in supplementary material Table S3. *Brachyury* and other T-Box morpholinos were validated previously (Gentsch et al., 2013). *Mixer* morpholino (sequence 5′-TTGGGAGCCACAAGCCTTGGGAACC-3′) was validated by inhibition of *in vitro* translation of HA-tagged *Mixer*, performed with TNT Transcription/Translation system and anti-HA western blot, using the TnT SP6 Quick Coupled Transcription/Translation System L2080 Kit (Promega). Morpholino (MO) injection levels were 20 ng per embryo for *Mixer* MO and the standard Gene Tools control MO, and 5 ng per embryo for each of four *Brachyury* morpholinos (Gentsch et al., 2013) to knock down the two paralogues *Brachyury* and *Brachyury-3*.

## Supplementary Material

Supplementary Material
