# Supplementary material for: High-resolution analysis of gene activity during the Xenopus mid-blastula transition
Source: Development. 2014 May;141(9):1927–39. doi: 10.1242/dev.102012 (PMC3994770; doi:10.1242/dev.102012)
Supplement: Supplementary Material [file supp_141_9_1927__index.html]

High-resolution analysis of gene activity during the Xenopus mid-blastula transition — Supplementary Material 

# High-resolution analysis of gene activity during the *Xenopus* mid-blastula transition

## DEV102012 Supplementary Material

**Files in this Data Supplement:**

- **Supplementary Material**
