# Supplementary material for: High-resolution analysis of gene activity during the Xenopus mid-blastula transition
Source: Development. 2014 May;141(9):1927–39. doi: 10.1242/dev.102012 (PMC3994770; doi:10.1242/dev.102012)
Supplement: Supplementary Material [file supp_141.9.1927_DEV102012.pdf]

Download Supp. Data File

Download Table S1

Download Table S2

Table S3. Sequences of primers used to analyse the expression of Mix1 intron sequences, Histone 1h4a, and Brachyury and Mixer targets

Gene	Forward primer 5′-3′	Reverse primer 5′-3′
Odc1	GTTCGACCTGCCAGAGCTAC	CAGGGAGAATGCCATGTTCT
Mix1 intron	GCACTGGCGGGTATAAAGAA	CCAAGCTTTGACCCATTAACA
Histone 1h4a	GAGTATGTCTGGACGCGGTA	TAGATGAGGCCAGAGATGCG
Plod2	TCATGATGCTTCAACATTCACA	ATGAAACTCCAGCCCTTCCT
Myf5	TGTGGATCGGATCTCCTCTT	TGTCAGGTGATCGTGGAAAG
Msgn	GAGAGGGAGAAGCTACGCAT	GGAGCAGGTTTGTGAGTTCA
Gata5	GATCCGCCGCCGTAATCCTG	ACCGCCCCTGGTTTGATT
Cer1	CCCACGCAAACAAAAGTTCAA	TGGCACCAGGCTTTTCAGTA
